# Hospitalizations Associated with COVID-19 Among Children and Adolescents — COVID-NET, 14 States, March 1, 2020–August 14, 2021

**DOI:** 10.15585/mmwr.mm7036e2

**Published:** 2021-09-10

**Authors:** Miranda J. Delahoy, Dawud Ujamaa, Michael Whitaker, Alissa O'Halloran, Onika Anglin, Erin Burns, Charisse Cummings, Rachel Holstein, Anita K. Kambhampati, Jennifer Milucky, Kadam Patel, Huong Pham, Christopher A. Taylor, Shua J. Chai, Arthur Reingold, Nisha B. Alden, Breanna Kawasaki, James Meek, Kimberly Yousey-Hindes, Evan J. Anderson, Kyle P. Openo, Kenzie Teno, Andy Weigel, Sue Kim, Lauren Leegwater, Erica Bye, Kathryn Como-Sabetti, Susan Ropp, Dominic Rudin, Alison Muse, Nancy Spina, Nancy M. Bennett, Kevin Popham, Laurie M. Billing, Eli Shiltz, Melissa Sutton, Ann Thomas, William Schaffner, H. Keipp Talbot, Melanie T. Crossland, Keegan McCaffrey, Aron J. Hall, Alicia M. Fry, Meredith McMorrow, Carrie Reed, Shikha Garg, Fiona P. Havers, COVID-NET Surveillance Team, COVID-NET Surveillance Team, Pam Daily Kirley, Sarah McLafferty, Isaac Armistead, Emily Fawcett, Katelyn Ward, Ruth Lynfield, Richard Danila, Sarah Khanlian, Kathy Angeles, Kerianne Engesser, Adam Rowe, Christina Felsen, Sophrena Bushey, Nasreen Abdullah, Nicole West, Tiffanie Markus, Mary Hill, Andrea George

**Affiliations:** ^1^CDC COVID-19 Response Team; ^2^Epidemic Intelligence Service, CDC; ^3^General Dynamics Information Technology, Atlanta, Georgia; ^4^California Emerging Infections Program, Oakland, California; ^5^Career Epidemiology Field Officer Program, CDC; ^6^University of California, Berkeley School of Public Health, Berkeley, California; ^7^Colorado Department of Public Health and Environment; ^8^Connecticut Emerging Infections Program, Yale School of Public Health, New Haven, Connecticut; ^9^Emory University School of Medicine, Atlanta, Georgia; ^10^Georgia Emerging Infections Program, Georgia Department of Health; ^11^Atlanta Veterans Affairs Medical Center, Atlanta, Georgia; ^12^Iowa Department of Health; ^13^Michigan Department of Health and Human Services; ^14^Minnesota Department of Health; ^15^New Mexico Emerging Infections Program, New Mexico Department of Health, Santa Fe, New Mexico; ^16^New Mexico Emerging Infections Program, University of New Mexico, Albuquerque, New Mexico; ^17^New York State Department of Health; ^18^University of Rochester School of Medicine and Dentistry, Rochester, New York; ^19^Rochester Emerging Infections Program, University of Rochester Medical Center, Rochester, New York; ^20^Ohio Department of Health; ^21^Public Health Division, Oregon Health Authority; ^22^Vanderbilt University Medical Center, Nashville, Tennessee; ^23^Salt Lake County Health Department, Salt Lake City, Utah; ^24^Utah Department of Health.; California Emerging Infections Program; Colorado Department of Public Health & Environment; Colorado Department of Public Health & Environment; Georgia Emerging Infections Program,; Georgia Department of Health and Foundation for Atlanta Veterans Education and Research, Decatur, Georgia, and Atlanta Veterans Affairs Medical Center, Atlanta, Georgia; Georgia Emerging Infections Program; Georgia Department of Health, and Division of Infectious Diseases, Emory University School of Medicine, Atlanta, Georgia; Minnesota Department of Health; Minnesota Department of Health; New Mexico Emerging Infections Program; New Mexico Emerging Infections Program; New York State Department of Health; New York State Department of Health; University of Rochester School of Medicine and Dentistry, Rochester, New York; University of Rochester School of Medicine and Dentistry, Rochester, New York; Public Health Division, Oregon Health Authority; Public Health Division, Oregon Health Authority; Vanderbilt University Medical Center, Nashville, Tennessee; Salt Lake County Health Department, Salt Lake City, Utah; Salt Lake County Health Department, Salt Lake City, Utah

Although COVID-19–associated hospitalizations and deaths have occurred more frequently in adults,^†^ COVID-19 can also lead to severe outcomes in children and adolescents (*1*,*2*). Schools are opening for in-person learning, and many prekindergarten children are returning to early care and education programs during a time when the number of COVID-19 cases caused by the highly transmissible B.1.617.2 (Delta) variant of SARS-CoV-2, the virus that causes COVID-19, is increasing.^§^ Therefore, it is important to monitor indicators of severe COVID-19 among children and adolescents. This analysis uses Coronavirus Disease 2019–Associated Hospitalization Surveillance Network (COVID-NET)^¶^ data to describe COVID-19–associated hospitalizations among U.S. children and adolescents aged 0–17 years. During March 1, 2020–August 14, 2021, the cumulative incidence of COVID-19–associated hospitalizations was 49.7 per 100,000 children and adolescents. The weekly COVID-19–associated hospitalization rate per 100,000 children and adolescents during the week ending August 14, 2021 (1.4) was nearly five times the rate during the week ending June 26, 2021 (0.3); among children aged 0–4 years, the weekly hospitalization rate during the week ending August 14, 2021, was nearly 10 times that during the week ending June 26, 2021.** During June 20–July 31, 2021, the hospitalization rate among unvaccinated adolescents (aged 12–17 years) was 10.1 times higher than that among fully vaccinated adolescents. Among all hospitalized children and adolescents with COVID-19, the proportions with indicators of severe disease (such as intensive care unit [ICU] admission) after the Delta variant became predominant (June 20–July 31, 2021) were similar to those earlier in the pandemic (March 1, 2020–June 19, 2021). Implementation of preventive measures to reduce transmission and severe outcomes in children is critical, including vaccination of eligible persons, universal mask wearing in schools, recommended mask wearing by persons aged ≥2 years in other indoor public spaces and child care centers,^††^ and quarantining as recommended after exposure to persons with COVID-19.^§§^

COVID-NET conducts population-based surveillance for laboratory-confirmed COVID-19–associated hospitalizations in 99 counties across 14 states^¶¶^ (*1*). Residents of the surveillance catchment area who received positive molecular or rapid antigen detection test results for SARS-CoV-2 during hospitalization or within 14 days before admission were classified as having COVID-19–associated hospitalizations. Unadjusted age-specific cumulative and weekly COVID-19–associated hospitalization rates (hospitalizations per 100,000 children and adolescents residing in the catchment area) during March 1, 2020–August 14, 2021, were calculated by dividing the total number of hospitalized patients by the National Center for Health Statistics’ population estimates within each age group for the counties included in the surveillance catchment area.*** Among adolescents, who are currently eligible for vaccination^†††^ (*3*), age-specific hospitalization rates during June 20–July 31, 2021, were calculated by COVID-19 vaccination status, which was determined for both hospitalized patients and the catchment area population using state immunization information systems data.^§§§^ Because the number of fully vaccinated persons in the underlying population changed weekly, incidence (cases per 100,000 person-weeks) was calculated by dividing the total number of vaccinated hospitalized adolescents by the sum of vaccinated adolescents in the underlying population each week; the same method was used to calculate incidence among unvaccinated adolescents.^¶¶¶^ Rate ratios and 95% confidence intervals (CIs) were calculated. Trained surveillance staff members conducted medical chart abstractions for all pediatric COVID-NET patients using a standardized case report form. Data on the following measures of severe disease were collected: median hospital length of stay, ICU admission, highest level of respiratory support received (i.e., invasive mechanical ventilation [IMV], bilevel positive airway pressure or continuous positive airway pressure, or high-flow nasal cannula), vasopressor use, and in-hospital death. Deaths occurring after hospital discharge were not included in this analysis. To assess COVID-19 severity among hospitalized children and adolescents in the setting of widespread Delta variant circulation, the proportions with measures of severe disease were compared between the periods before (March 1, 2020–June 19, 2021) and after (June 20–July 31, 2021) the Delta variant became the predominant strain circulating in the United States**** (*4*). A Wilcoxon rank sum test was used to compare medians; chi square or Fisher’s exact tests were used to compare proportions. Data were analyzed using SAS (version 9.4; SAS Institute); statistical significance was defined as p<0.05. This activity was reviewed by CDC and was conducted consistent with applicable federal law and CDC policy.^††††^

During March 1, 2020–August 14, 2021, COVID-NET identified 49.7 cumulative COVID-19–associated hospitalizations per 100,000 children and adolescents (Figure 1); rates were highest among children aged 0–4 years (69.2) and adolescents aged 12–17 years (63.7) and lowest among children aged 5–11 years (24.0). Weekly hospitalization rates were at their lowest in 2021 during the weeks ending June 12–July 3 (0.3 per 100,000 children and adolescents each week) (Figure 2). During a subsequent 6-week period after the Delta variant became predominant, rates rose each week to 1.4 during the week ending August 14, 2021, which was 4.7 times the rate during the week ending June 26, 2021 and approached the peak hospitalization rate of 1.5 observed during the week ending January 9, 2021.^§§§§^ Weekly rates increased among all age groups; the sharpest increase occurred among children aged 0–4 years, for whom the rate during the week ending August 14, 2021 (1.9) was nearly 10 times that during the week ending June 26, 2021 (0.2). During June 20–July 31, 2021, among 68 adolescents hospitalized with COVID-19 whose vaccination status had been ascertained, 59 were unvaccinated, five were partially vaccinated, and four were fully vaccinated; the hospitalization rate among unvaccinated adolescents was 0.8 per 100,000 person-weeks (95% CI = 0.6–0.9), compared with 0.1 (95% CI = 0.0–0.1) in fully vaccinated adolescents (rate ratio = 10.1; 95% CI = 3.7–27.9).

**FIGURE 1:**
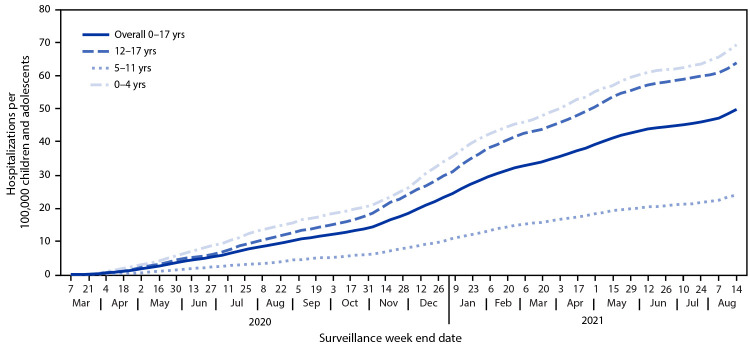
COVID-19–associated cumulative hospitalizations per 100,000 children and adolescents,* by age group — COVID-NET, 14 states,^†^ March 1, 2020–August 14, 2021 * Rates are subject to change as additional data are reported. ^†^ Select counties in California, Colorado, Connecticut, Georgia, Iowa, Maryland, Michigan, Minnesota, New Mexico, New York, Ohio, Oregon, Tennessee, and Utah.

**FIGURE 2:**
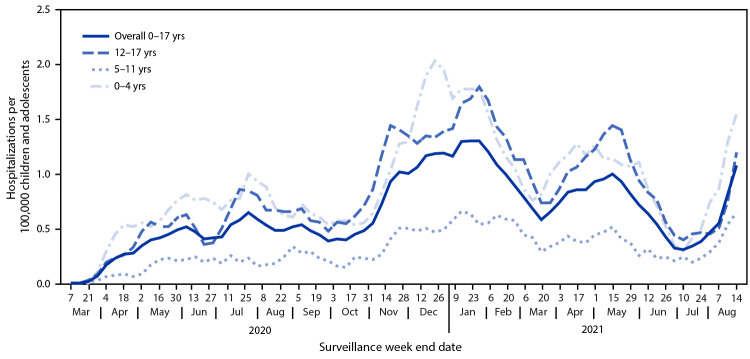
COVID-19–associated weekly hospitalizations per 100,000 children and adolescents,* by age group — COVID-NET, 14 states,^†^ March 1, 2020–August 14, 2021 (3-week smoothed running averages)^§^ * Rates are subject to change as additional data are reported. ^†^ Select counties in California, Colorado, Connecticut, Georgia, Iowa, Maryland, Michigan, Minnesota, New Mexico, New York, Ohio, Oregon, Tennessee, and Utah. ^§^ Smoothed running averages are used for visualization purposes only.

Among 3,116 hospitalized children and adolescents with COVID-19 during March 1, 2020–June 19, 2021, for whom complete clinical data were available,^¶¶¶¶^ 827 (26.5%) were admitted to an ICU, 190 (6.1%) required IMV, and 21 (0.7%) died. Among 164 hospitalized children and adolescents with COVID-19 during June 20–July 31, 2021, for whom complete clinical data were available,***** 38 (23.2%) were admitted to an ICU, 16 (9.8%) required IMV, and three (1.8%) died. The differences in these indicators of severe disease between the two periods were not statistically significant (Table).

**TABLE:** Clinical interventions and outcomes among children and adolescents aged 0-17 years during COVID-19–associated hospitalizations — COVID‑NET, 14 states,* March 1, 2020–June 19, 2021 and June 20–July 31, 2021

Interventions and outcomes	Children and adolescents hospitalized, No. (%)		p-value^§^
	March 1, 2020–June 19, 2021 (N = 3,116)^†^	June 20–July 31, 2021 (N = 164)^†^	
**Hospital length of stay, median (interquartile range)**	3 (2–5)	2 (1–4)	0.01
**Outcome**			
Died during hospitalization	21 (0.7)	3 (1.8)	0.12
**ICU admission**	827 (26.5)	38 (23.2)	0.34
**Vasopressor support**	233 (7.5)	13 (7.9)	0.83
**Highest level of respiratory support** ^¶^			
High flow nasal cannula	162 (5.2)	13 (7.9)	0.13
BiPAP/CPAP	131 (4.2)	6 (3.7)	0.73
Invasive mechanical ventilation	190 (6.1)	16 (9.8)	0.06

**Abbreviations:** BiPAP = bilevel positive airway pressure; CPAP = continuous positive airway pressure; ICU = intensive care unit.

* Select counties in California, Colorado, Connecticut, Georgia, Iowa, Maryland, Michigan, Minnesota, New Mexico, New York, Ohio, Oregon, Tennessee, and Utah.

^†^ Includes those with complete clinical data on hospital length of stay, ICU admission, highest level of respiratory support (invasive mechanical ventilation, BiPAP/CPAP, or high flow nasal cannula), vasopressor support, and disposition discharge (i.e., discharged alive or died in-hospital).

^§^ Medians were compared using a Wilcoxon rank sum test. Proportions were compared using chi square tests. The proportions who died during hospitalization were compared using Fisher’s exact test.

^¶^ Highest level of respiratory support for each patient that needed respiratory support.

## Discussion

Weekly COVID-19–associated hospitalization rates rose rapidly during late June to mid-August 2021 among U.S. children and adolescents aged 0–17 years; by mid-August, the rate among children aged 0–4 years was nearly 10 times the rate 7 weeks earlier. This increase coincides with widespread circulation of the highly transmissible Delta variant. COVID-NET data indicate that vaccination was highly effective in preventing COVID-19–associated hospitalizations in adolescents during late June to late July 2021. Since March 2020, approximately one in four hospitalized children and adolescents with COVID-19 has required intensive care, although the proportions with indicators of severe disease during the period when the Delta variant predominated were generally similar compared with those earlier in the pandemic. The observed indicators of severe COVID-19 among children and adolescents, as well as the potential for serious longer-term sequelae (e.g., multisystem inflammatory syndrome in children) documented elsewhere (*5*,*6*), underscore the importance of implementing multipronged preventive measures to reduce severe COVID-19 disease, including nonpharmaceutical interventions and vaccination among eligible age groups.^†††††^

Among adolescents aged 12–17 years, the only pediatric age group for whom a COVID-19 vaccine is currently approved, hospitalization rates were approximately 10 times higher in unvaccinated compared with fully vaccinated adolescents, indicating that vaccines were highly effective at preventing serious COVID-19 illness in this age group during a period when the Delta variant predominated. As of July 31, 2021, 32% of U.S. adolescents had completed a COVID-19 vaccination series (*7*); increasing vaccination coverage among adolescents, as well as expanding eligibility for COVID-19 vaccination to younger age groups if approved and recommended, is expected to reduce severe COVID-19–associated outcomes among children and adolescents.

Similar to another recent analysis, COVID-NET data suggest that indicators of severe disease among hospitalized children during an early period when the Delta variant predominated were generally similar to those observed earlier in the pandemic (*8*). Trends in outcomes will need to be monitored closely as more data become available. For example, whereas the point estimate of the proportion of hospitalized children who required IMV during the period of Delta predominance (9.8%) was higher than that earlier in the pandemic (6.1%), the comparison of these proportions was based on a relatively small number of children (16) requiring IMV during the period of Delta predominance, and the difference was not statistically significant (p = 0.06). Further, surveillance data limited to hospitalized persons cannot be used to assess whether increases in COVID-19–associated hospitalization rates among children and adolescents are due to increased community SARS-CoV-2 transmission or increased disease severity caused by the Delta variant.

The findings in this report are subject to at least five limitations. First, children and adolescents meeting COVID-NET criteria with a positive SARS-CoV-2 test result might have been hospitalized primarily for reasons other than COVID-19 (*2*), resulting in potential overestimations of hospitalization rates. Second, COVID-19–associated hospitalizations might have been missed because of testing practices and test availability. Third, the number of hospitalized children with severe outcomes was small during June 20–July 31, 2021, limiting comparisons between periods before and during Delta variant predominance. Fourth, the number of fully vaccinated hospitalized adolescents remained low at the time of reporting, and hospitalization rates stratified by vaccination status are subject to error if misclassification of vaccination status occurred. Finally, the COVID-NET catchment areas include approximately 10% of the U.S. population; thus, findings might not be nationally generalizable.

Rates of COVID-19–associated hospitalization among children and adolescents increased rapidly from late June to mid-August 2021, coinciding with predominance of the Delta variant. With more activities resuming, including in-person school attendance and a return of younger children to congregate child care settings, preventive measures to reduce the incidence of severe COVID-19 are critical. Universal indoor masking is recommended for all teachers, staff members, students, and visitors in kindergarten through grade 12 schools, regardless of vaccination status.^§§§§§^ CDC recommends that persons aged ≥2 years who are unvaccinated, as well as vaccinated persons in areas of substantial or high transmission, wear masks in all indoor public spaces.^¶¶¶¶¶^ CDC also recommends that child care centers serving children too young to be vaccinated consider implementing universal indoor masking for persons aged ≥2 years.****** All persons who are eligible should receive COVID-19 vaccines to reduce the risk for severe disease for themselves and others with whom they come into contact, including children who are currently too young to be vaccinated.

**Summary**

**What is already known about this topic?**

COVID-19 can cause severe illness in children and adolescents.

**What is added by this report?**

Weekly COVID-19–associated hospitalization rates among children and adolescents rose nearly five-fold during late June–mid-August 2021, coinciding with increased circulation of the highly transmissible SARS-CoV-2 Delta variant. The proportions of hospitalized children and adolescents with severe disease were similar before and during the period of Delta predominance. Hospitalization rates were 10 times higher among unvaccinated than among fully vaccinated adolescents.

**What are the implications for public health practice?**

Preventive measures to reduce transmission and severe outcomes in children and adolescents are critical, including vaccination, universal masking in schools, and masking by persons aged ≥2 years in other indoor public spaces and child care centers.

## Footnotes

* These authors contributed equally to this report.

†
https://www.cdc.gov/coronavirus/2019-ncov/covid-data/investigations-discovery/hospitalization-death-by-age.html

§
https://www.cdc.gov/coronavirus/2019-ncov/variants/index.html

¶
https://www.cdc.gov/coronavirus/2019-ncov/covid-data/covid-net/purpose-methods.html

** COVID-NET hospitalization data are preliminary and subject to change as more data become available. In particular, case counts and rates for recent hospital admissions are subject to lag.

††
https://www.cdc.gov/coronavirus/2019-ncov/prevent-getting-sick/about-face-coverings.html

§§
https://www.cdc.gov/coronavirus/2019-ncov/your-health/quarantine-isolation.html

¶¶ California, Colorado, Connecticut, Georgia, Iowa, Maryland, Michigan, Minnesota, New Mexico, New York, Ohio, Oregon, Tennessee, and Utah. The included counties have been listed previously. https://www.cdc.gov/mmwr/volumes/69/wr/mm6932e3.htm

*** Rates are calculated using the National Center for Health Statistics’ vintage 2019 bridged-race postcensal population estimates for the counties included in surveillance (https://www.cdc.gov/nchs/nvss/bridged_race.htm).

†††
https://www.cdc.gov/coronavirus/2019-ncov/vaccines/recommendations/adolescents.html

§§§ The Food and Drug Administration granted emergency use authorization for the Pfizer-BioNTech COVID-19 vaccine for adolescents aged 12–15 years on May 10, 2021. The earliest date that adolescents in this age group could have met the definition for being a fully vaccinated patient hospitalized with COVID-19 was June 14, 2021. Because vaccination data are subject to lag, rates by vaccination status were only calculated through July 31, 2021.

¶¶¶ Fully vaccinated adolescents with COVID-19–associated hospitalizations were defined as those who had received a second vaccine dose ≥14 days before a positive SARS-CoV-2 test result associated with their hospitalization. Adolescents whose positive SARS-CoV-2 test date was ≥14 days after a single dose through <14 days after a second dose were considered partially vaccinated and were not included in rates; adolescents who had received a single dose of vaccine <14 days before the positive SARS-CoV-2 test result were also not included in rates. If the SARS-CoV-2 test date was not available, hospital admission date was used. Adolescents whose vaccination status had not yet been verified using the immunization information system data were considered to having missing vaccination status and were excluded. Adolescents whose vaccination status was checked against the immunization information system and who did not have documented receipt of any vaccine dose before the test date were considered unvaccinated. Additional COVID-NET methods for determining vaccination status have been described previously. https://medrxiv.org/cgi/content/short/2021.08.27.21262356v1

****
https://covid.cdc.gov/covid-data-tracker/#variant-proportions

†††† 45 C.F.R. part 46.102(l)(2); 21 C.F.R. part 56; 42 U.S.C. Sect. 241(d); 5 U.S.C. Sect. 552a; 44 U.S.C. Sect. 3501 et seq.

§§§§ Smoothed 3-week running averages are used for visualization purposes in Figure 2; however, raw (nonaveraged) age-specific weekly rates are used in the text of the report. The smoothed 3-week running average rate of COVID-19–associated hospitalizations during the week ending January 9, 2021 (displayed in Figure 2) is 1.3 hospitalizations per 100,000 children and adolescents.

¶¶¶¶ Among the 3,194 children and adolescents with COVID-19–associated hospitalizations during March 1, 2020–June 19, 2021, a total of 3,116 (97.6%) had data available on hospital length of stay, ICU admission, receipt of IMV or other respiratory support, vasopressor use, and in-hospital death at the time of reporting.

***** Among the 191 children and adolescents with COVID-19–associated hospitalizations during June 20, 2020–July 31, 2021, a total of 164 (85.9%) had data available on hospital length of stay, ICU admission, receipt of IMV or other respiratory support, vasopressor use, and in-hospital death at the time of reporting.

†††††
https://www.cdc.gov/vaccines/hcp/acip-recs/vacc-specific/covid-19.html

§§§§§
https://www.cdc.gov/coronavirus/2019-ncov/community/schools-childcare/k-12-guidance.html

¶¶¶¶¶
https://www.cdc.gov/coronavirus/2019-ncov/vaccines/fully-vaccinated.html

******
https://www.cdc.gov/coronavirus/2019-ncov/community/schools-childcare/child-care-guidance.html
